# Risk of endometrial, ovarian, and breast cancers in women with polycystic ovary syndrome: A systematic review and meta-analysis

**DOI:** 10.18502/ijrm.v20i11.12357

**Published:** 2022-12-10

**Authors:** Mina Amiri, Razieh Bidhendi-Yarandi, Aida Fallahzadeh, Zahra Marzban, Fahimeh Ramezani Tehrani

**Affiliations:** ^1^Reproductive Endocrinology Research Center, Research Institute for Endocrine Sciences, Shahid Beheshti University of Medical Sciences, Tehran, Iran.; ^2^Department of Biostatistics, University of Social Welfare and Rehabilitation Sciences, Tehran, Iran.; ^3^School of Medicine, Tehran University of Medical Science, Tehran, Iran.

**Keywords:** Polycystic ovary syndrome, Endometrial cancer, Ovarian cancer, Breast cancer.

## Abstract

**Background:**

Although several studies have evaluated the risk of gynecological cancers in women with polycystic ovary syndrome (PCOS), there are controversies regarding it.

**Objective:**

This study aimed to investigate the association of PCOS with endometrial, ovarian, and breast cancers.

**Materials and Methods:**

PubMed, Scopus, Web of Science, and Google Scholar databases based on MESH terms using the combination of the appropriate keywords were searched to retrieve observational studies on endometrial, ovarian, and breast cancers in PCOS women, published from inception to April 2020. This meta-analysis was performed to determine the pooled odds ratio (OR) of these cancers in women with PCOS. Publication bias was assessed by using Begg's test.

**Results:**

Of 1347 records retrieved by searching the databases, a total of 14 articles were included in the study. Overall, the pooled OR of the composite outcome, including endometrial, ovarian, and breast cancers in women with PCOS was higher than that of women with no PCOS (pooled OR: 1.4, 95% CI: 1.0-1.9). The pooled OR of endometrial (pooled OR: 2.2, 95% CI: 1.03-4.7) and ovarian (pooled OR: 1.3, 95% CI: 1.0-1.8) cancers in women with PCOS was higher than the control group, whereas the pooled OR of breast cancer was not significantly higher than that of the control group.

**Conclusion:**

This meta-analysis indicated an increased risk of endometrial and ovarian cancers in women with PCOS.

## 1. Introduction 

Polycystic ovary syndrome (PCOS), one of the common abnormalities in reproductive age women (1), is defined by ovulation abnormalities, high levels of androgens, and polycystic ovaries in ultrasonography (2). PCOS is also associated with several conditions, such as glucose intolerance, diabetes, hypertension, central obesity, metabolic syndrome, and cardiovascular diseases (3-7).

The high prevalence of endometrial hyperplasia and carcinoma due to chronic anovulation, associated with prolonged exposure to unopposed estrogen has been long recognized. Moreover, PCOS complications, such as obesity, nulliparity, diabetes, and hypertension, are risk factors for endometrial carcinoma (8-10). In this regard, a population-based cohort study reported a 17-fold increase in the risk of endometrial cancer among women with PCOS, compared to those without PCOS (11). Several hypotheses have been proposed for the increased risk of ovarian cancer in PCOS women, such as anovulation, increased androgen exposure, and lack of progesterone (12). Another study found that the risk of ovarian cancer was 2.5 times higher in women with PCOS compared to healthy women (13). It is possible that the sustained elevation of serum estrogen levels may lead to the growth of hormone-sensitive tumors, such as breast cancer (14); therefore, anovulation can be considered as a critical risk factor in women with PCOS. Several studies have investigated the risk of breast cancer in women with PCOS; however, the actual risk remains unclear (11, 15-17). Although some studies have assessed the risk of endometrial, ovarian, and breast cancers in women with PCOS, their results are often conflicting, and the risk of these cancers in women with PCOS is still debated (11, 13, 15, 16, 18).

Hence, this study aims to conduct a meta-analysis of observational studies to investigate the association of PCOS with endometrial, ovarian, and breast cancers. Since the severity of PCOS manifestations can distort the results, we adjusted the results for PCOS diagnostic criteria in a meta-regression analysis.

## 2. Materials and Methods

This meta-analysis was designed according to the guidelines for the preferred reporting items for systematic reviews and meta-analyses (PRISMA) to assess the pooled odds ratio of endometrial cancer in women with PCOS, compared to healthy controls (19).

### Search strategy 

Data was searched using PubMed, Scopus, Web of Science, and Google Scholar for retrieving studies published up to April 2020 investigating gynecological cancers in women with PCOS.

Two reviewers (M.A. and A.F.) performed searches separately. Search on PubMed was performed initially, based on MESH terms using the following keywords: (`polycystic ovary syndrome' OR `PCOS' OR `Stein-Leventhal Syndrome') AND (`breast cancer' OR `breast tumor' OR `breast neoplasm' OR `breast carcinoma' OR `Human Mammary Carcinoma' OR `ovarian cancer' OR `ovarian neoplasm' OR `carcinoma' OR `ovarian epithelial' OR `ovary cancer' OR `cancer of ovary' OR `ovarian tumor' OR `endometrial neoplasm' OR `endometrial carcinoma' OR `endometrial cancer' OR `cancer of the endometrium' OR `cancer, endometrium' OR `carcinoma, endometrial' OR `endometrial tumor').

Search limitations were humans and English language publications. There was no time limitation. The same search strategy was applied for all databases, based on the titles, abstracts, and keywords. We applied a `pearl growing' strategy. First, we obtained the full text of the studies, then the reference list of the studies was reviewed to prevent missing related articles.

### Eligibility criteria

All types of observational studies, including case-control, cross-sectional, and cohort designs were eligible to be included in the meta-analysis. Studies needed to report raw data of events, odds ratio (OR), and relative risk to provide sufficient information to allow calculation. Any PCOS diagnosis criteria were eligible to be included, for example, Rotterdam, National Institute of Health, Androgen Excess Society, International Classification of Diseases, and also a self-reported questionnaire.

The exclusion criteria included studies 1) assessing conditions, for example, polycystic ovary, androgen excess disease instead of PCOS, 2) without control groups, 3) with unreliable and incomplete results, and 4) assessing hyperplasia rather than cancer.

### Study selection 

All relevant studies assessing at least one of the cancers of endometrial, ovarian, and breast in women with PCOS were included in this meta-analysis.

Search results were screened based on the predefined eligibility criteria. All references were entered into EndNote software. The initial selection was based on article titles, and then a second selection based on abstracts was done and duplicates were deleted by one reviewer (A.F). Finally, the full text of selected articles was reviewed for data extraction. Disagreements were resolved by 2 other reviewers (M. A. and F.R.T.).

### Data extraction

Two reviewers (M.A. and A.F.) extracted data from the full text of selected articles and checked twice to lessen errors. For each study, information such as author's name, publications year, article title, design of the study, study population, number of outcomes, and unadjusted or adjusted OR, relative risk, or HR of the outcomes were extracted.

### Quality assessment

M.A. and A.F. assessed the quality of the included studies and F.R.T. resolved any disagreements. Newcastle-Ottawa scale was applied for the quality assessment (20).

The high quality was defined when a study got ≥ 70% of the highest level of the Newcastle-Ottawa scale, moderate quality was defined when a study got 40-70%, and low quality was defined when a study got 20-40% and those with < 20% were defined as very low quality.

### Bias assessment

Cochrane collaboration's tools were applied to assess the risk of bias in each study (21).

### Outcome measures 

In this meta-analysis, outcomes of interest were endometrial cancer, ovarian cancer, and breast cancer.

### Statistical analysis

This meta-analysis was conducted to obtain a pooled OR of endometrial, ovarian, and breast cancer in PCOS women. Heterogeneity was assessed via I-squared statistics and in the case of values with upper limits 50% random effect method was applied, otherwise the fixed-effect method.

To assess publication bias, Begg's test was used (22); it was found significant for p < 0.05, and in this case trim and fill method was conducted to correct for publication bias by adding some study measures (23, 24). A funnel plot was also drawn to depict publication bias issues. A Forest plot was also drawn to summarize the result of each study's effect sizes and its 95% confidence intervals.

In case of publication bias, forest plot corrected via trim and fill method was drawn. To estimate pooled ORs, we applied the `Meta-prop' random effect method (25). The 95% prediction interval (95% PI) was estimated to evaluate clinical significance as compared to statistical significance for the pooled OR. Moreover, the random effect meta-regression model was fitted to assess the effects of PCOS diagnosis criteria and age on the results. We also run a sensitivity analysis to detect probable influential studies with a high risk of bias. Statistical analysis was conducted using STATA software (version 13; STATA, INC., college station, TX, USA).

## 3. Results

### Search results, study selection, study characteristics, and quality assessment

**Figure 1:**
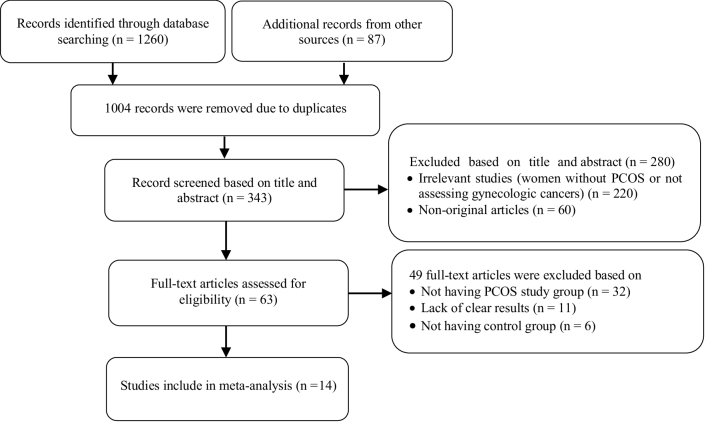
Flow diagram of the study.

Figure 1 presents the flow diagram of the search strategy and study selection. Of 1347 records retrieved by searching the databases, a total of 14 studies, including 4 cohorts (11, 15, 17, 26) and 10 case-control (13, 16, 17, 27-34) studies, were selected for the final analysis. Nine studies (11, 13, 15-17, 26-28, 30, 34) were classified as high-quality, and 5 (17, 29, 31-33) as moderate-quality (Table I-II).

Two studies (11, 15) based on the international classification of diseases, 2 (16, 29) used the Rotterdam criteria, 1 (26) used the laparoscopic criteria, and 9 (13, 17, 27, 28, 30-34) reported no criteria. In addition, 7 (11, 15-17, 26, 29, 32) reported breast cancer, 6 (11, 13, 15, 27, 30, 33) reported ovarian cancer, and 6 (11, 15, 26, 28, 31, 34) reported endometrial cancer.

### Meta-analysis and meta-regression of outcomes

In this meta-analysis, 12,955 women with PCOS and 118,481 controls were included. The pooled mean (95% CI) of age and body mass index of all study populations were 27.8 yr (95% CI: 27.8-27.9) and 26.5 kg/m^2^ (95% CI: 26.3-26.7), respectively. Table III presents the results of the meta-analysis and meta-regression.

**Figure 2:**
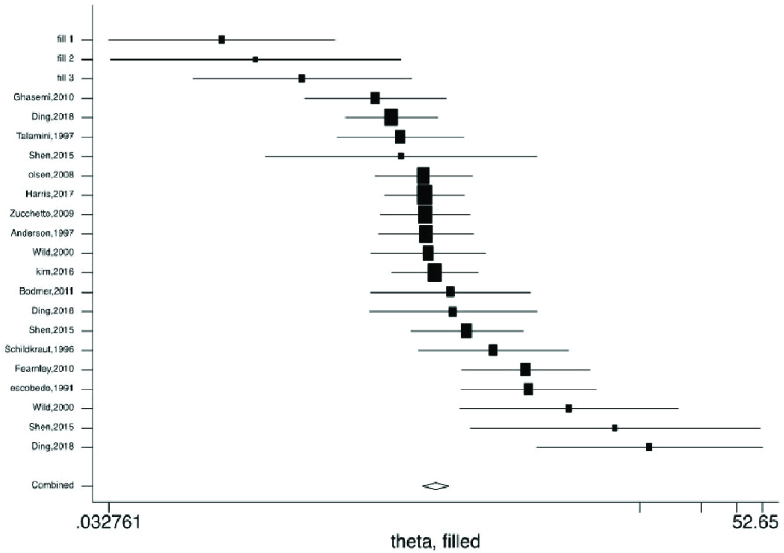
Forest plots by trim and fill method for the overall outcome. ◊: Pooled estimation of odds ratio (OR) with random effect method.

**Figure 3:**
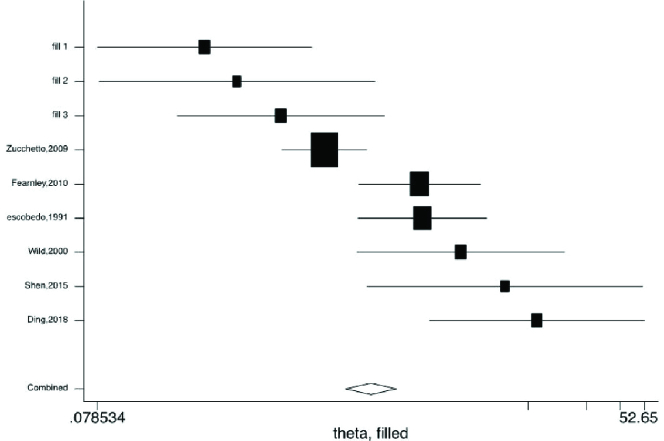
Forest plots by trim and fill method for endometrial cancer. ◊: Pooled estimation of odds ratio (OR) with random effect method.

**Figure 4:**
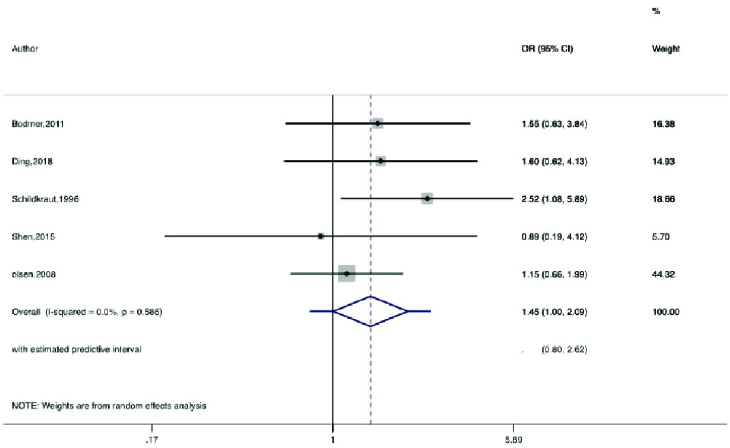
Forest plot for ovarian cancer. ◊: Pooled estimation of odds ratio (OR) with random effect method.

**Figure 5:**
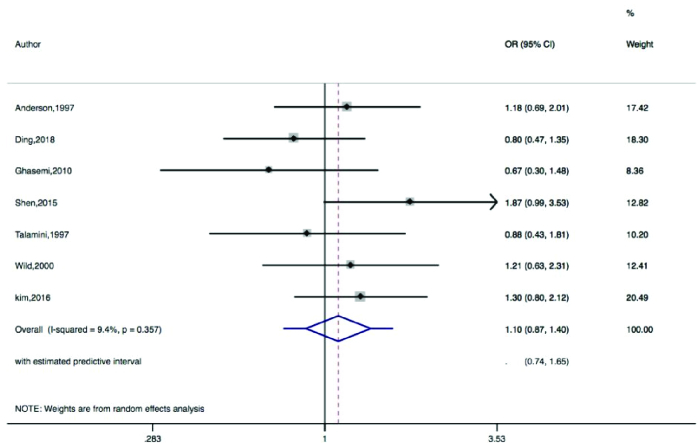
Forest plot for breast cancer. ◊: Pooled estimation of odds ratio (OR) with random effect method.

Figures 2-5 present the forest plots of the pooled ORs for the composite outcome (overall outcome) as well as separate outcomes, including endometrial, ovarian, and breast cancers in women with PCOS vs. the control group. The pooled OR of the composite outcome in women with PCOS was higher than that of women with no PCOS (pooled OR: 1.4, 95% CI: 1.0-1.9). The results also indicated that the pooled OR of endometrial and ovarian cancers in women with PCOS was higher than that of their counterparts (pooled OR: 2.2, 95% CI: 1.03-4.7 and pooled OR: 1.3, 95% CI: 1.0-1.8, respectively), whereas the pooled OR of breast cancer in women with PCOS was not significantly higher than controls.

**Figure 6:**
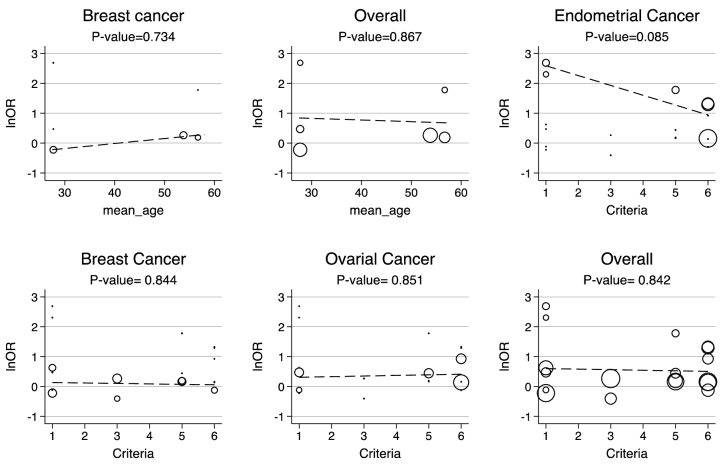
Bubble plots to display heterogeneity among the risk of cancers in levels of mean age and PCOS diagnosis criteria for sufficient observation, the p-value has been obtained from random effect meta-regression analysis.

Moreover, the 95% PIs for the odds of endometrial cancer, ovarian cancer, breast cancer, and all cancer types were estimated in women with PCOS vs. their counterparts (95% PI: 0.33-9.5; 95% PI: 0.74-1.65; 95% PI: 0.89-1.98; and 95% PI: 0.57-4.5, respectively). The results of meta-regression analysis revealed that the diagnostic criteria of PCOS and age had no significant effects on the heterogeneity of the outcomes (Figure 6).

### Publication bias, risk of bias, and sensitivity analysis

**Figure 7:**
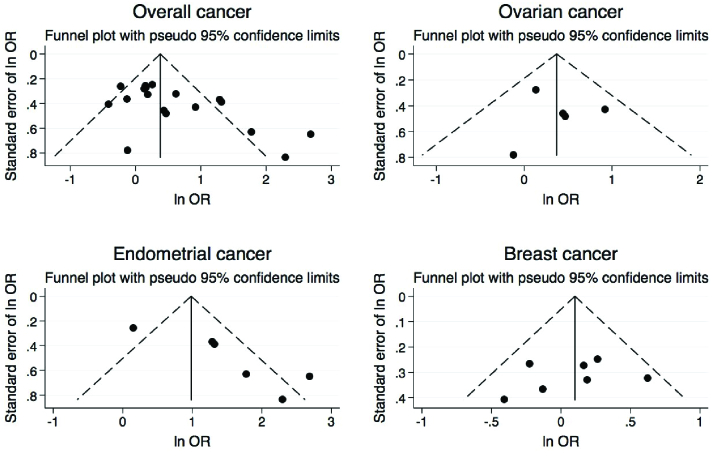
Funnel plots for endometrial, ovarian, breast cancer, and overall outcomes.

The results of Begg's test showed a significant publication bias for endometrial cancer in both PCOS and non-PCOS groups and breast cancer in the PCOS group, which was adjusted by the trim and fill method (Table IV, Figure 7).

**Figure 8:**
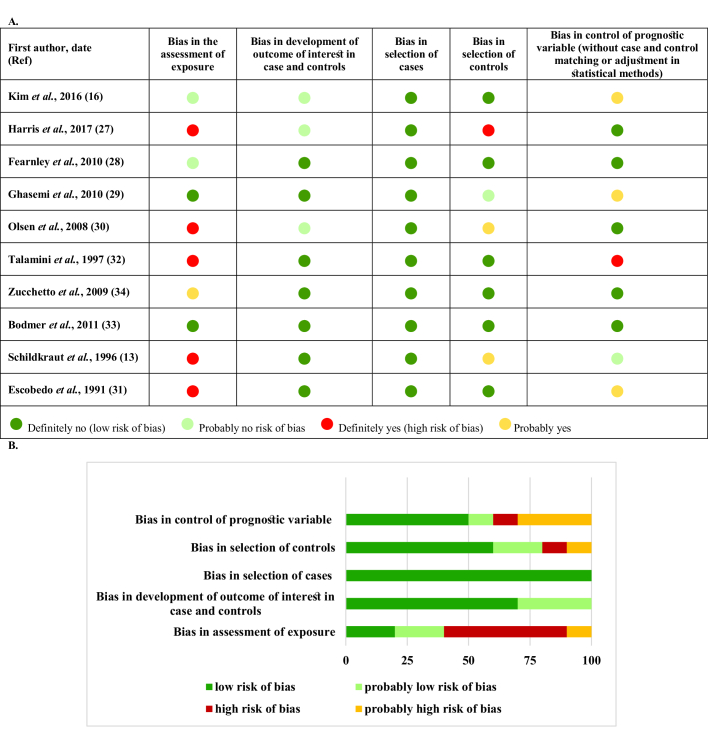
Risk of bias in case-control studies. A) Risk of bias summary B) Risk of bias graph.

**Figure 9:**
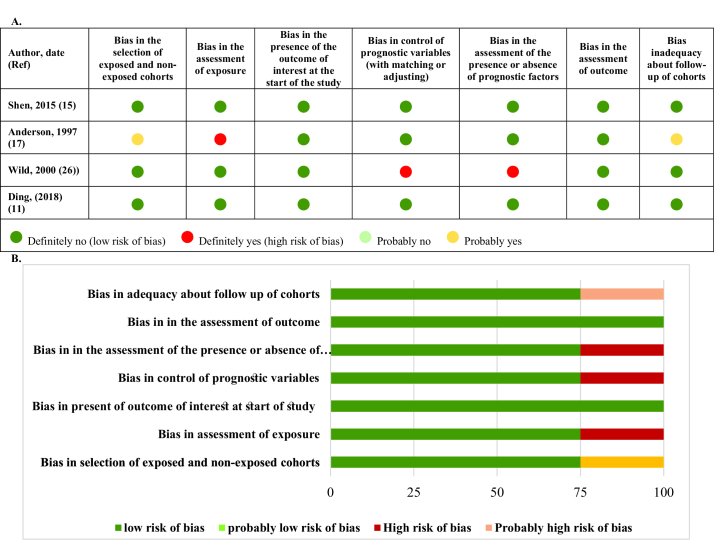
Risk of bias in cohort studies. A) Risk of bias summary. B) Risk of bias graph.

Figures 8-9 represent the risk of bias in the included studies. Most case-control studies had a low risk of bias in domains of sample selection and the primary outcome in the case and control groups, despite a high risk of bias in the assessment of exposure and control of the prognostic variable. In cohort studies, there was a low risk of bias in the adequacy of follow-ups, assessment of outcomes, selection of exposed and non-exposed cohorts, and presence of the outcome of interest at the onset of the study. Moreover, we found a high risk of bias in controlling for prognostic variables, assessment of exposure, and assessment of the presence or absence of prognostic factors. The sensitivity analysis of endometrial cancer, ovarian cancer, breast cancer, and all cancers showed that no study caused heterogeneity among the results (Figure 10).

**Table 1:** The results of quality assessment of case-control studies


	**Selection**				**Comparability**		**Exposure**		**Total scores**
**Author, year (Ref) **	**Adequate case definition**	**Representativeness of the cases**	**Community selection of controls**	**No history of disease among controls**	**A: Study controls for age and/or BMI B: Study control for any additional factors**	**A: Secure record for clinical outcome B: Structured interview were blind to case/control status**	**The same method of ascertainment for cases and controls**	**Same nonresponse rate for cases and controls**	
	**Fearnley, 2010 (28)**	*	*	*	*	**	*	*	–	7
**Ghasemi, 2010 (29)**	*	–	–	*	–	*	*	–	4
**Talamini, 1997 (32)**	*	–	*	*	–	*	*	*	6
**Schildkraut, 1996 (13)**	*	*	*	*	**	*	*	*	9
**Bodmer, 2011 (33)**	*	*	*	*	–	–	*	*	6
**Olsen, 2008 (30)**	*	*	*	*	**	–	*	*	8
**Zucchetto, 2009 (34)**	*	*	*	*	**	*	*	*	9
**Harris, 2017 (27)**	*	*	*	*	**	–	*	*	8
**Kim, 2016 (16)**	*	*	*	*	**	–	*	–	7
**Escobedo, 1991 (31)**	*	*	–	–	*	*	*	*	6
The quality assessment has been evaluated based on the Newcastle-Ottawa scale. A study can be awarded a maximum of one star (*) for each numbered item within the selection and exposure categories. A maximum of 2 stars can be given for comparability, BMI: Body mass index									

**Table 2:** The results of quality assessment of cohort studies


	**Selection**				**Comparability**	**Outcome**			**Total scores**
**Author, year (Ref)**	**Representativeness of the exposed cohort**	**Selection of the nonexposed cohort**	**Ascertainment of exposure**	**No outcome of interest at the start of the study**	**A: Study controls for age and/or BMI, B: Study controls for other confounders**	**A: Doctor's diagnosis OR objective measurements, B: Parent/self-reported doctor's diagnosis OR use of medication**	**Follow-up long enough for outcomes (at least 10 yr)**	**Adequacy of follow-up of cohorts**	
	**Shen, 2015 (15)**	*	*	*	*	**	*	*	*	9
**Ding, 2018 (11)**	*	*	*	*	**	*	*	*	9
**Anderson, 1997 (17)**	–	*	–	*	**	*	–	*	6
**Wild, 2000 (26)**	*	*	*	*	–	*	*	*	7
The quality assessment has been evaluated based on the Newcastle-Ottawa scale. A study can be awarded a maximum of one star (*) for each numbered item within the selection and exposure categories. A maximum of 2 stars can be given for comparability, BMI: body mass index									

**Table 3:** The results of meta-analysis, meta-regression, and publication bias of studies conducted on the prevalence / OR of gynecologic cancer in PCOS patients


**Outcomes**	**Number of observations**	**^#^I^2^%**	**^&^Publication bias**	**^£^Pooled OR (95%CI), (95%PI)**	**^@^Meta-regression **
**Endometrial cancer**	${}^{\$}$ 6+3	> 50	0.015*	^1^Insufficient observations
**PCOS**	${}^{\$}$ 7+3	> 50	0.004*	
**NON-PCOS**	${}^{\$}$ 6+3	> 50	0.039*	${}^{\$}$ 2.2 (1.03, 4.7)* (0.33, 9.5)	^2^0.71 (0.10), 62.89%
**Ovarian cancer**	6	< 50	0.950			
**PCOS**	7	> 50	0.764		Insufficient observations
**NON-PCOS**	5	> 50	0.051	1.3 (1.0, 1.8)* (0.74, 1.65)		1.0 (0.07), 0.00%
**Breast cancer**	7	< 50	0.452		1.0 (0.10), 0.00%
**PCOS**	${}^{\$}$ 8+4	> 50	0.015*		
**NON-PCOS**	7	> 50	0.652	1.1 (0.87, 1.4) (0.89, 1.98)	0.98 (0.09), 23.98%
**Overall**	${}^{\$}$ 19+3	> 50	0.045*			0.99 (0.07), 82.46%
**PCOS**	${}^{\$}$ 22+10	> 50	0.000*		
**NON-PCOS**	${}^{\$}$ 18+9	> 50	0.000*	${}^{\$}$ 1.4 (1.0, 1.9)* (0.57,4.5)	0.98 (0.09), 68.29%
${}^{\#}$ I-square (I^2^) was used to assess heterogeneity, ${}^{\&}$ Beggs' test was run to assess publication bias, *Significant level was considered at p < 0.05, ${}^{\$}$ Trim and fill correction method was applied, ${}^{£}$ To estimate pooled odds ratios (ORs), we applied the “Meta-prop” random effect method. The 95% prediction interval (95% PI) was estimated to evaluate clinical significance as compared to statistical significance for the pooled odds ratio, ${}^{@}$ Exponential (beta regression coefficient) (Std. Err., I-squared residuals %): 1- age adjusted, 2- PCOS diagnostic criteria adjusted, PCOS: Polycystic ovary syndrome					

**Table 4:** Characteristics of studies included in the meta-analysis


**Author, yr (Ref)**	**Country**	**Study design**	**PCOS diagnostic criteria**	**Cancer diagnostic criteria**	**PCOS group characteristics**	**Control group characteristics**	**Outcome of interest**	**Adjustments**	**Unadjusted OR** **(95% CI)**	**Quality assessment**
**Ding ** ***et al.*** **, 2018 (11)**	Taiwan	Cohort	ICD-9-CM^1^ Code 256.4	ICD-9-CM Code 174 and 175	N = 8155 Age = 27.7 (7.0) BMI = Not reported	N = 32620 Age = 27.5 (7.9) BMI = Not reported	Endometrial, ovarian, and breast cancer	Age, comorbidities	Endometrial cancer OR: 14.685 (4.096, 52.650) ovarian cancer OR:1.60 (0.62, 4.13) breast cancer OR:0.80 (0.47, 1.35)	High
**Harris ** ***et al.*** **, 2017 (27)**	US	Case-control	Not reported	Not reported	N = 78 Age and BMI not reported for case	N = 4063 Age and BMI not reported for control	Ovarian cancer	Age, study, parity, oral contraceptive use, center, tubal ligation, family history of ovarian cancer	1.16 (0.74, 1.82)	High
**Kim ** ***et al.*** **, 2016 (16)**	US	Case-control	Rotterdam criteria	Not reported	N = 67 Age = 53.8 ± 12.2 BMI = 27.4 ± 6.2	N = 2951 Age = 58.0 ± 12.8 BMI = 26.5 ± 5.7	Breast cancer	Age, physical activity	1.30 (0.80, 2.12)	High
**Shen ** ***et al.*** **, 2015 (15)**	Taiwan	Cohort	NIH^4^ or ICD-9-CM	ICD-9-CM Code 174	N = 3566 Age = 27.04 (22.66-32.10) BMI = Not reported	N = 14264 Age = 27.05 (22.72-32.10) BMI = Not reported	Endometrial, ovarian, and breast cancer	Age, hypertension, diabetes mellitus, dyslipidemia, coronary artery disease, congestive heart disease, cerebrovascular disease, chronic pulmonary disease, urbanization, and income	Endometrial cancer OR: 10.013 (1.942, 51.629) ovarian cancer OR: 0.89 (0.19, 4.12) breast cancer OR: 1.87 (0.99, 3.53)	High
**Ghasemi ** ***et al.*** **, 2012 (29)**	Iran	Case-control	Rotterdam	Histologically confirmed	N = 27 Age = 30-51 BMI not reported	N = 305 Age = 30-51 BMI not reported	Breast cancer	None	0.67 (0.30, 1.48)	Moderate
**Bodmer ** ***et al.*** **, 2011 (33)**	UK	Case-control	Used diagnosis from GPRD	Based on evidence of cancer-related therapy	N = 28 Age = 61.2 ± 13.1	N = 10753 Age = 61.2 ± 13.1	Ovarian cancer	BMI, smoking, prior, use of hormones, history o hysterectomy, endometriosis	1.55 (0.63, 3.84)	Moderate
**Fearnley ** ***et al.*** **, 2010 (28)**	Australia	Case-control	Not reported	Histologically confirmed	N = 32 Age < 50 yr BMI = not reported	N = 522 Age < 50 yr BMI = Not reported	Endometrial cancer	BMI	3.638 (1.761, 7.515)	High
**Zuchetto ** ***et al.*** **, 2009 (34)**	Italy	Case-control	Not reported	Histologically confirmed	N = 68 Age = 18-79 BMI not reported	N = 1287 Age = 18-79 BMI not reported	Endometrial cancer	Period of interview, BMI, age at menarche, age at menopause, parity, and oral contraceptive use	1.171 (0.706, 1.943)	High
**Olsen ** ***et al.*** **, 2008 (30)**	Australia	Case-control	Not reported	Histologically confirmed	N = 52 Age = 18-79 BMI not reported	N = 3047 Age = 18-79 BMI not reported	Ovarian cancer	Age, education, parity, hormonal contraceptive use, and BMI	1.15 (0.66, 1.99)	High
**Wild ** ***et al.*** **, 2000 (26)**	UK	Cohort	Laparoscopic	ICD-7 and ICD-8	N = 319 Age = 38-98 BMI = 27.1	N = 1060 Age = 38-98 BMI = 26.2	Endometrial and breast cancer	BMI	Endometrial cancer OR: 5.923 (1.721, 20.365) breast cancer OR: 1.21 (0.63, 2.31)	High
**Anderson ** ***et al.*** **, 1997 (17)**	USA	Cohort	Not reported	ICD codes 174.0-174.9	N = 472 Age = 55-69 BMI = 27.2 ± 0.23	N = 34363 Age = 55-69 BMI = 27 ± 0.03	Breast cancer	Age, age at menarche, age at first pregnancy, parity, oral contraceptive use, hormone replacement therapy, BMI, waist to hip ratio, benign breast disease, and family history of breast cancer	1.18 (0.69, 2.01)	Moderate
**Talamini ** ***et al.*** **, 1997 (32)**	Italy	Case-control	Not reported	Histologically confirmed	N = 30 Age = 20-74 BMI not reported	N = 5127 Age = 20-74 BMI not reported	Breast cancer	Age at menarche	0.88 (0.43, 1.81)	Moderate
**Schildkraut ** ***et al.*** **, 1996 (13)**	USA	Case-control	Not reported	Histologically confirmed	N = 31 Age = 20-54 BMI not reported	N = 4526 Age = 20-54 BMI not reported	Ovarian cancer	Age, parity, and oral contraceptive use	2.52 (1.08, 5.89)	High
**Escobedo ** ***et al.*** **, 1991 (31)**	Georgia	Case-control	Not reported	Histologically confirmed	N = 30 Age = 20-54 BMI not reported	N = 3593 Age = 20-54 BMI not reported	Endometrial cancer	Age	3.757 (1.747, 8.081)	Moderate
PCOS: Polycystic ovary syndrome, BMI: Body mass index, N: Number, OR: Odds ratio, ICD: International classification of disease, NIH: National institute of health, ICD-9-CM: International classification of diseases, ninth revision, clinical modification, GPRD: General practice research database										

**Figure 11:**
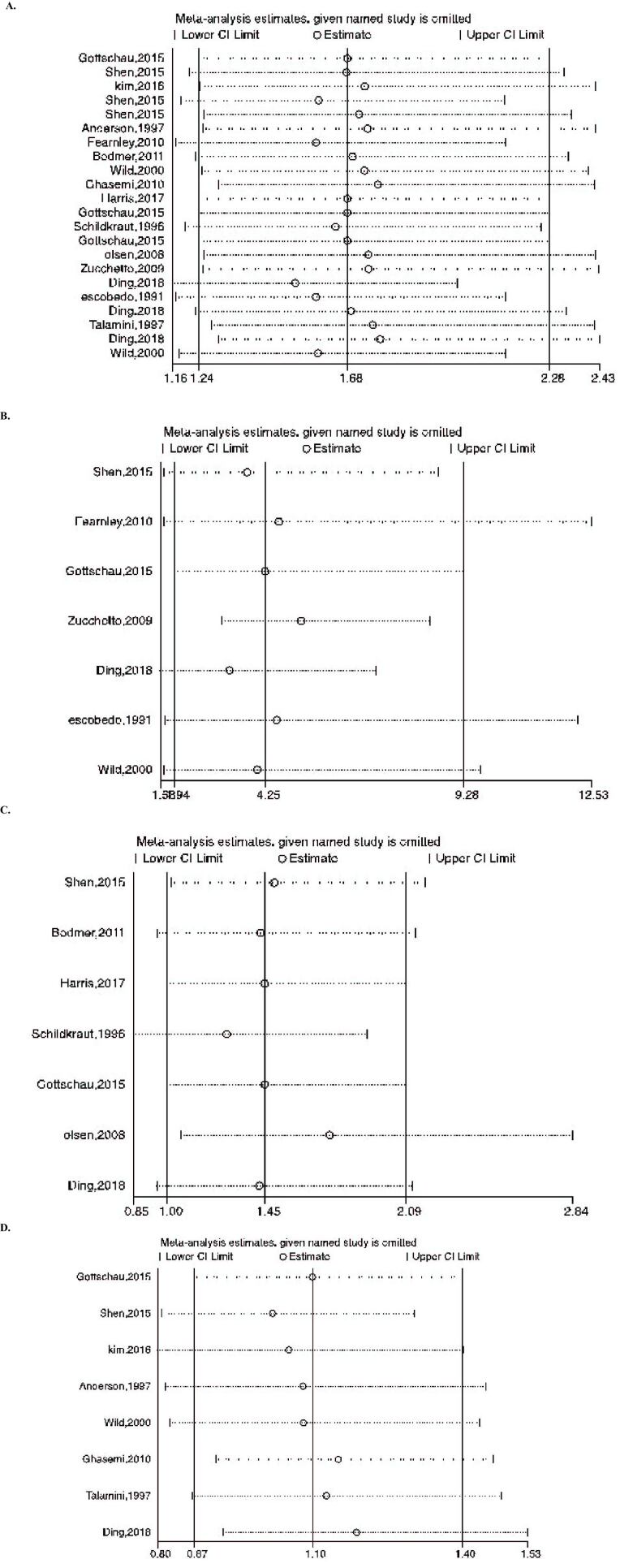
Results of sensitivity analysis to present influential studies for A) Overall, B) Endometrial, C) Ovarian, and D) Breast cancers, respectively.

## 4. Discussion

This meta-analysis was carried out to assess the association of PCOS with endometrial, ovarian, and breast cancers. The results revealed that PCOS was associated with an increased risk of endometrial and ovarian cancers, but not breast cancer.

It is known that in women with PCOS who have anovulatory menstrual cycles, progesterone, a key hormone in the endometrium against estrogen-driven growth, does not play a regulatory role. This can result in the development of endometrial hyperplasia and adenocarcinoma, mainly due to constant unopposed estrogen activity in the endometrium (35). In other words, an estrogen/progesterone imbalance may lead to endometrial hyperplasia increasing the risk of endometrial cancer in the long run (36). Also, the endometrium of women with PCOS, who received ovulation induction showed the downregulation of progesterone-regulated genes in the secretory phase, leading to progesterone resistance (37). Overall, hyperandrogenism is a clinical feature of PCOS (2). Besides, secretion of ovarian steroids, such as testosterone, is higher in women with endometrial cancer, compared to the healthy population (38). It is well documented that women with PCOS have increased levels of endometrial androgen receptors, compared to the fertile controls (39). Hypersecretion of luteinizing hormone is another feature of PCOS (40). This finding is important since the expression of luteinizing hormone receptors increase in women with anovulatory cycles and endometrial hyperplasia, and it may be associated with endometrial carcinogenesis (41). Insulin resistance is another common feature in many women with PCOS, which leads to compensatory hyperinsulinemia and a 4-fold increase in the prevalence of type II diabetes (40, 42). Hyperinsulinemia, caused by insulin resistance, promotes endometrial cell proliferation and increases the risk of endometrial cancer (14). Also, evidence suggests that the risk of endometrial cancer is higher in women with diabetes compared to those without diabetes (43). Other risk factors for endometrial cancer, such as obesity, nulliparity, and hypertension, are also associated with PCOS (14).

Our results showed that the risk of endometrial cancer was 2.2 times higher in women with PCOS than the controls. In agreement with our findings, a meta-analysis of 4 studies suggested that women with PCOS were 3 times more likely to develop endometrial cancer, compared to those without PCOS (14). Likewise, another meta-analysis of 5 studies showed a 3-fold increase in the risk of endometrial cancer in PCOS women compared to the general population (44). Similarly, a meta-analysis of 11 studies demonstrated that the risk of endometrial cancer was nearly 3 times higher in PCOS women compared to their counterparts (45).

It is well-documented that ovulation is an etiological cause of ovarian cancer; however, it cannot be the only contributor to the pathogenesis of this cancer (12). Although the risk of ovarian cancer in women with PCOS is expected to be low due to anovulation, it is higher than that of healthy women (6). Hormonal mechanisms are hypothesized to be involved in the etiology of ovarian cancer (46). Increased androgen exposure in women with PCOS has been hypothesized to be associated with an increased risk of ovarian cancer (12). This association could be explained by evidence regarding the presence of androgen receptors on healthy ovarian cells, as well as benign and borderline tumors (12). Moreover, it has been shown that higher androgen levels during pregnancy were associated with an increased risk of borderline serous and mucinous tumors (47, 48). Hyperinsulinemia and the resulting increase in insulin-like growth factor-1, which plays an important role in tumorigenesis, have also been suggested as the main mechanism (49).

The present study indicates that the risk of ovarian cancer is 1.3 times higher in PCOS women than their counterparts. In line with our results, a meta-analysis conducted in 2009 (14) reported that women with PCOS are twice more likely to develop ovarian cancer, compared to those without PCOS. Our results are also consistent with the findings reported by another meta-analysis, which showed a 2.5-fold increase in the risk of ovarian cancer in women with PCOS aged < 54 yr, compared to those with no PCOS; however, no significant association was detected between PCOS and ovarian cancer before excluding women aged over 54 yr (45).

There is a complex relationship between PCOS and breast cancer, as the consequences of PCOS have been associated with both the increased and decreased risk of breast cancer (18). The anovulatory cycle and infertility are among the characteristics of PCOS, suggested to decrease the risk of breast cancer (50). However, obesity is a major risk factor for breast cancer in both post and premenopausal women, and as mentioned previously, it is also a common finding in PCOS women (51). This reduction in the risk of breast cancer among women with ovulatory disorders is because of luteal phase deficiency in the menstrual cycle; therefore, the levels of estrogen and progesterone do not increase. Also, it is well-documented that breast cancer cell proliferation is higher during the luteal phase (18, 52). Moreover, hyperinsulinemia has been suggested as an independent risk factor for breast cancer (53). The correlation between androgen excess and the pathogenesis of PCOS is still controversial; however, androgens seem to trigger the development of estrogen-receptor (ER)-negative breast cancer (54, 55). Advanced maternal age during the first pregnancy and nulliparity are also important risk factors for breast cancer (56).

Despite the mentioned mechanisms, we found no significant association between PCOS and breast cancer. Similar to our results, a meta-analysis showed that women with PCOS were not exposed to a higher risk of breast cancer, compared to those without PCOS (14). Likewise, another meta-analysis of 8 studies found no significant association between PCOS and breast cancer (57). Similarly, a recent meta-analysis reported no significant association between PCOS and breast cancer (45). However, more comprehensive prospective cohort studies are needed to examine the association between PCOS and breast cancer and to identify the involved mechanisms.

In our meta-analysis, the risk of bias assessment revealed the high risk of bias for some included studies, especially case-control studies, which might influence our results. To detect these possible effects, we performed a sensitivity analysis. However, no significant studies were found, which could cause heterogeneity.

The most important strength of our study was including a larger number of studies, especially cohort studies. Unlike previous meta-analyses, we did not include studies investigating women with polycystic ovarian morphology, who were not diagnosed with PCOS (58-60); this may increase the accuracy of our findings.

On the other hand, the main limitation of this meta-analysis was that a large number of studies did not report the mean age and details of diagnostic criteria for PCOS. However, after considering the PCOS diagnostic criteria and the mean age through meta-regression, we found that these variables were not significant sources of heterogeneity. Also, in most previous studies, cases of self-reported PCOS have effects on the exactness and validity of the results. Even though in this meta-analysis we tried to lessen all possible biases, it should be considered that there was significant heterogeneity in most of the outcomes. Since this heterogeneity could be due to variations in PCOS phenotypes and diagnostic criteria, we considered the results for PCOS diagnostic criteria via meta-regression. However, our findings showed that the diagnostic criteria of PCOS exerted no significant effects on the heterogeneity of the outcomes.

Moreover, a large number of studies, identified through our database search, did not have any control groups, which could limit the number of studies eligible for our analysis. While some potential confounders, such as body mass index, might affect the results, we could not consider these variables due to the paucity of data. Although pooling fully adjusted ORs can provide more real effect sizes, we considered unadjusted ORs, since included studies had not adjusted the same confounders that may lead to further bias for summary effect size in meta-analyses, especially for weak or medium associations so that the direction of causal inference would be even reversed (61). Also, we could not consider family history, lifestyle, medication use (e.g., metformin or oral contraceptive pills), and many other conditions, which can play important roles in the development of cancer. Ovarian cancer consists of distinct histotypes; however, we combined them due to the paucity of data. Moreover, considering the borderline statistical significance of the results, we estimated the clinical significance by measuring PIs, which showed no clinical significance, possibly due to the limited number of included studies. All of these limitations should be considered in interpreting the results.

## 5. Conclusion

This study indicated the increased risk of endometrial and ovarian cancers in women with PCOS. Therefore, screening programs for early detection of these cancers, especially in women with PCOS, can be considered an important strategy for improving their survival.

##  Conflict of Interest

The authors have no conflict of interest to declare.
